# A case of recalcitrant hidradenitis suppurativa concomitantly treated with tirzepatide

**DOI:** 10.1016/j.jdcr.2024.02.023

**Published:** 2024-03-06

**Authors:** Lina J. Chan, Manjit Kaur, Benjamin H. Kaffenberger

**Affiliations:** a Division of Research, Kirk Kerkorian School of Medicine at University of Nevada, Las Vegas, Las Vegas, Nevada; b Department of Dermatology, The Ohio State University Wexner Medical Center, Columbus, Ohio

**Keywords:** gastric inhibitory peptide/glucagon-like peptide 1 agonist, GLP-1, hidradenitis suppurativa, tirzepatide

## Introduction

Hidradenitis suppurativa (HS) is a chronic, autoinflammatory cutaneous, and systemic disease manifesting as deep, painful nodules in apocrine gland-bearing regions, with accompanying fistula formation, resulting in malodor and scarring.^1^ This multifactorial disease intertwines genetic, environmental, and immunologic factors, and is often complicated by comorbidities. HS is often associated with comorbidities such as obesity which has deleterious effects on the quality of life (QOL) of these patients and contributes to the heightened disease burden in this population. Notably, obesity is prevalent in up to 75% of patients with HS, contributing to a systemic inflammatory state and a higher body mass index has been positively correlated with disease severity.^2^^,^^3^ In a retrospective study involving 35 patients with substantial weight reduction after bariatric surgery, 70% had improvement or complete cessation of HS flares.^4^ Although approved treatment options remain limited, an expanding range of adjunct therapies for HS management are emerging, including weight reduction strategies with glucagon-like peptide 1 (GLP-1) agonists.^5^ Additionally, the dual incretin coagonist, tirzepatide, is a combined gastric inhibitory peptide/GLP-1 agonist which are key mediators for insulin secretion as well as regulatory mechanisms in the brain for satiety. Tirzepatide has a higher affinity for gastric inhibitory peptide receptors, however, both peptides increase insulin production and stimulate glucose effectiveness.^6^ This promising molecule has had head-on trials with semaglutide, a GLP-1 agonist, with tirzepatide groups dosed at 5, 10, and 15 mg achieving a statistically significant reduction in glycated hemoglobin levels from baseline with a difference of –0.15 (*P* = .02), –0.39 (*P* < .001), and –0.45 (*P* < .001) respectively compared with semaglutide group at 40 weeks.^7^ Five head-on clinical trials (SURPASS 1-5) compared tirzepatide to semaglutide, with tirzepatide reducing glycosylated hemoglobin (1.24%-2.58%) and body weight (5.4-11.7 kg) by unmatched values compared with a single GLP-1 agonist. Additionally, up to 62.4% of patients reported in these studies have had a reduction in glycosylated hemoglobin <5.7%.^8^

## Case report

A female patient in her 20s with a 10-year history of HS, associated with polycystic ovarian disease, type 2 diabetes mellitus, and obesity (body mass index 41.36 kg/m^2^) presented after failing topicals and systemics, including antibiotics, oral contraceptives, spironolactone, oral corticosteroids, and adalimumab. She presented with inflammatory, painful nodules, and draining sinus tracts in her axillae, inframammary region, and inguinal folds. She is a nonsmoker with no family history of HS.

She was initially treated with infliximab and reported notable improvement in pain, suppuration, and malodor. Eight months later, she initiated tirzepatide (2.5 mg/0.5 mL once a week), and at this point had a calculated Dermatology Life Quality Index score of 14, visual analog scale of 3, and Hidradenitis Suppurativa Physician’s Global Assessment of 3, indicating moderate severity. Tirzepatide was increased to (7.5 mg/0.5 mL once a week) over a period of 3 months for diabetes with obesity. She continued to experience occasional boils, albeit with reduced inflammation. Her calculated Dermatology Life Quality Index decreased from 14 (significantly affecting her life) to 3 (no effect on her life), and visual analog scale from 3 to 1. An improvement in HS was exhibited by Hidradenitis Suppurativa Physician’s Global Assessment change from 3 (moderate) to 2 (mild) and Hidradenitis Suppurativa Clinical Response, with a decrease in the number of lesions from baseline 1 abscess to 0, and 4 baseline inflammatory nodules to 3 with no draining fistulas. Posttherapy, she had improved glycemic control and lipid profile (Table I). Additionally, she successfully lost 16% of her body weight 3 months after starting tirzepatide.

**Table I:** Patient’s glycemic laboratory tests, body mass index, patient reported, and physician reported outcomes before and after tirzepatide therapy

	Before tirzepatide	3 mo after tirzepatide
Fasting blood sugar	179	95
Random blood sugar	220	108
HbA1C	10.1	5.4
Triglycerides	233	139
BMI	41.36	33.63
DLQI	14	3
VAS	3	1
HS-PGA	Moderate	Mild
HiSCR-sum of abscesses and inflammatory nodules	5	3

*BMI*, Body mass index; *DLQI*, Dermatology Life Quality Index; *HbA1C*, glycosylated hemoglobin; *HiSCR*, Hidradenitis Suppurativa Clinical Response; *HS-PGA*, Hidradenitis Suppurativa Physician’s Global Assessment; *VAS*, visual analog scale.

## Discussion

Hidradenitis suppurativa can be debilitating for patients significantly affecting their QOL because of the recurrent, intractable nature of the disease, chronic pain, and associated scarring. The disease presents a therapeutic challenge, and obesity contributes to poor health and impaired QOL of patients with HS, negatively contributing to the significant impact of the disease itself.^5^ Despite our patient receiving infliximab infusions and reporting a robust response, weight reduction, and glycemic control achieved with the use of tirzepatide, concomitantly with infliximab, in this case, led to a significant enhancement in QOL and Hidradenitis Suppurativa Clinical Response. This case underscores the innovative application of dual incretin coagonists for weight reduction in obese, diabetic HS patients with moderate to severe chronic disease, a strategy dermatologists may consider adding to their repertoire alongside biologics. However, further studies are warranted to determine its efficacy for the treatment of moderate to severe HS associated with comorbidities such as obesity with or without diabetes.

## Conflicts of interest

None disclosed.
